# Sexual and gender diversity in nursing: radicalizing knowledge and practices

**DOI:** 10.1590/1980-220X-REEUSP-2025-0275en

**Published:** 2026-03-02

**Authors:** Lucas Cardoso dos Santos, Aurea Tamami Minagawa Toriyama, Lucia Yasuko Izumi Nichiata

**Affiliations:** 1 Universidade de São Paulo, Escola de Enfermagem, Departamento de Enfermagem em Saúde Coletiva, São Paulo, SP, Brazil.; 2 Universidade de São Paulo, Escola de Enfermagem, Departamento de Enfermagem Materno-Infantil e Psiquiátrica, São Paulo, SP, Brazil.

This perspective proposes a reflection on the need to radicalize nursing knowledge and practices in the face of sexual and gender diversity, highlighting how processes of invisibility, silencing, and erasure are present in the field of health. Such radicalization should not be understood as a rupture devoid of dialogue, but as a deepening of nursing’s ethical-political and scientific commitment to social justice. It is based on a critical perspective capable of challenging exclusionary norms. Furthermore, it defends the urgency of advancing knowledge and consolidating health practices that not only include but also redress historical injustices.

Sexual and gender diversity has been understood in different ways throughout human history, and has emerged in contemporary debates as an object of analysis by various researchers and social movements.

Gender diversity encompasses distinct identity possibilities beyond “feminine” and “masculine”, including other ways of representing and socially performing gender expressions^(1)^. Thus, gender identity is a person’s self-perception of gender and how they express it to society. A person whose gender identity corresponds to the sex assigned at birth can be called cisgender. However, if a person does not have a gender identity equivalent to the sex assigned at birth, they may be understood as a transgender or non-binary person^(1,2)^, among other identities that deviate from the sex-gender system imposed at birth^(3)^.

Sexual diversity, in turn, encompasses the plurality of experiences and manifestations of human sexuality^(1)^. Each person’s experiences regarding sexual orientation are interpreted as the direction of physical, romantic, and sexual attraction towards a specific gender, or as being independent of gender and gender identity, as well as the absence (or little) of attraction^(1,2)^. Sexual and affective orientations can occur between people of the same gender (homosexuality), between different genders with which a person identifies (heterosexuality), between both genders (bisexuality), and can also be characterized by the absence or low level of sexual attraction (asexuality), among other manifestations. This reveals that human sexuality is neither fixed nor uniform, but rather traversed by multiple ways of feeling, desiring, and relating.

Both definitions refer to gender and sexual diversity, the plurality of the social, of relationships, and of the coexistence of different characteristics, perspectives, and experiences within a group and society. They are precisely the opposite of cisheteronormativity, a term that arose from the need to analyze and critique the idea that heterosexuality and identification with the gender assigned at birth are the social norm^(4)^. Understanding these concepts as synonyms and adopting a cisheteronormative meaning in social relations imposes violence in various social processes, especially on people who have sexual orientations and gender identities that diverge from traditional social norms^(5,6)^.

In Brazil, the country where the most lesbian, gay, bisexual, transgender, queer, intersex, asexual, pansexual, nonbinary and other gender identities and sexual orientations (LGBTQIAPN+) people are murdered in the world, with one death every 34 hours^(7,8)^, approximately 19 million people identify as belonging to this group, accounting for 12% of the general population^(6)^. However, official data on this population are not found in different countries, revealing that the statistical invisibility of the LGBTQIAPN+ population is, in itself, an expression of social determination, as it prevents the recognition of this group’s health needs.

In light of this scenario, LGBTQIAPN+ people face worse working and living conditions, as well as higher rates of violence, negatively impacting their access to education and formal employment and, consequently, their health needs^(6,7)^. Thus, the ways in which the LGBTQIAPN+ population becomes ill and dies are profoundly historically and socially determined. The inequities experienced by the LGBTQIAPN+ population, such as stigma, exclusion, violence, and denial of rights—expressions of social determination—operate as structural elements that produce health vulnerabilities^(1,2,6,7)^.

At this point, it is important to note that the people who make up the LGBTQIAPN+ acronym are not a homogeneous group in their health needs, but rather form an identity group that contradicts traditional definitions of heterosexuality and cisgenderism, experiencing the historical logic of exclusion and silencing of dissident bodies. Addressing the injustices imposed on this group requires acknowledging the historical debt owed to this population, built over centuries of pathologization, marginalization, absence, and violence. This stance requires more than inclusion: it requires active reparation, with affirmative actions that confront the legacy of exclusion, promoting these individuals’ leading role in the construction of public policies and the guarantee of rights.

The Brazilian National Policy for Comprehensive Health of Lesbians, Gays, Bisexuals, Transvestites and Transsexuals, enacted in 2011, constitutes one of the reparative measures within the scope of the Brazilian Health System (In Portuguese, *Sistema Único de Saúde* - SUS) to guarantee rights, such as access to healthcare services, inclusive education, and protection against violence and prejudice. It presents an operational plan with strategies to address health inequities, structured around four pillars: “access to comprehensive healthcare for the Lesbian, Gay, Bisexual, Transvestite and Transgender (LGBT) population”; “health promotion and surveillance actions for the LGBT population”; “continuing education and popular health education focused on the LGBT population”; “monitoring and evaluation of health actions for the LGBT population”^(9)^.

The implementation of this policy is contingent upon coordinated action by governments at different levels, including state and municipal departments, health councils, and bodies of the Ministry of Health. Furthermore, the active presence of civil society, in its various forms of organization, is an indispensable element for ensuring rights, reducing inequalities, and promoting the democratic exercise of social control. However, the effectiveness of this policy is still limited by a lack of awareness and weak monitoring and evaluation mechanisms that hinder the identification of gaps, the adjustment of practices, and the full inclusion of the LGBTQIAPN+ community in healthcare services^(10,11)^. Therefore, there is a need to strengthen strategies that articulate funding, professional training, and intersectoral actions, ensuring that the principles of universality, comprehensiveness, and equity of the SUS are concretely applied to all gender identities and sexual orientations.

LGBTQIAPN+ individuals have historically suffered prohibitions, constraints, and violence in healthcare services at the hands of students and healthcare professionals, resulting in the spread of stigma and prejudice, the exclusion of this population from services, and an increase in health inequities, revealing significant gaps in healthcare training^(12)^. The themes of sexual and gender diversity have been marginalized and/or given little emphasis in Brazilian health curricula^(13)^. Nursing, in particular, the largest healthcare workforce in Brazil and worldwide, due to its strategic position in healthcare work, has a fundamental— and still largely unexplored—social role in building inclusive, ethical, and humanized practices aimed at LGBTQIAPN+ people^(5,12,14,15,16,17)^.

In the United States of America, the time dedicated to teaching about the LGBTQIAPN+ people’s specific needs in university nursing courses is estimated at two hours throughout the entire training^(15)^. In Brazil, this scenario is repeated, where the approach to health issues related to sexual and gender diversity still occurs marginally, if not absent^(5,16)^. This curricular omission contributes to the perpetuation of exclusionary practices and to the naturalization of cisheteronormativity in training spaces.

In graduate studies, where most Brazilian nursing research is produced, a review of dissertations and theses on the LGBTQIAPN+ population’s health, covering the period from 2011 to 2022, showed that most studies sought to elucidate issues regarding this population’s access to healthcare services. Barriers were identified as a result of prejudice, violence, lack of qualified assistance, and difficulties in accessing information for disease prevention and treatment. The study also revealed a lack of research authored by LGBTQIAPN+ individuals from social movements and a need for research that advances the applicability of nursing theories in the care of this population^(17)^.

The findings highlight the urgent need to align training, research, and care practices with the LGBTQIAPN+ population’s real needs. In this regard, it is essential that the field of health education critically and transversally incorporates the themes of sexual and gender diversity at its different levels, with the explicit objective of implementing inclusive, affirmative, and restorative health practices capable of challenging exclusionary norms. In university extension activities, privileged spaces should be provided for closer ties between the university and the community, fostering dialogue, active listening, and the collective construction of care practices that are more sensitive to diversity. In clinical practice, it is necessary to invest in strategies for raising awareness and providing continuing training for healthcare teams. In nursing research, it is essential to contribute to the production of knowledge about LGBTQIAPN+ individuals’ health needs. It is imperative that research supports practices from a perspective that values the inclusion and empowerment of the LGBTQIAPN+ population.

Successful experiences in nursing education have shown significant progress when sexual and gender diversity is systematically incorporated. The use of active methodologies and the cross-curricular inclusion of content, coupled with the participation of LGBTQIAPN+ individuals in training processes, contribute to expanding practices that are more representative and sensitive to this population’s needs and to enhancing students’ competencies^(18,19)^. Within the institutional context, explicit inclusion policies and leaders’ engagement in training and service reorganization processes have fostered healthier work environments and improved quality of care^(20,21)^. Such initiatives demonstrate that the critical and consistent inclusion of these themes not only enhances training but also transforms care and work contexts, reaffirming nursing’s ethical and political commitment.

Therefore, it is argued that nursing work is intrinsically political in nature, as it involves decision-making, resource mobilization, and influence on health policies, with the aim of guaranteeing access to and quality of care for individuals and communities. Nursing, comprised of political agents of transformation, must actively combat institutional prejudice against LGBTQIAPN+ people, promoting safe environments, advocating for health policies that address the specific needs of this population, and transforming the realities of bodies that deviate from the norm.

## FINAL CONSIDERATIONS

From this perspective, it is argued that nursing care can only be truly comprehensive and equitable when it is based on respect for individuals’ uniqueness and active listening to their voices. This should guide the development of policies, research, knowledge, teaching, and care practices as a path to effective transformation. Combating prejudice and acknowledging the historical debt of exclusion are fundamental steps towards justice and reparation.

Sexual and gender diversity as an essential dimension of nursing work must be recognized as an ethical, political, and professional imperative. Overcoming historical gaps in training, confronting prejudices still present in care practices, and proposing new ways of caring requires commitment, recognition, and respect for professional duties, especially in the understanding that gender identity and sexual orientation are important elements of the health-disease process.

Expanding nursing practice beyond the biomedical dimension at different levels of healthcare and education, due to its widespread reach and constant presence among individuals, families, and communities, plays a strategic role with transformative and unique potential in this process. Consolidating a practice that recognizes, respects, and celebrates sexual and gender diversity is, ultimately, reaffirming the fundamental values of respect for human dignity, commitment to life, and the struggle for a more just, pluralistic, and diverse society. Therefore, the importance of nursing participation in the formulation of more inclusive public policies and the systematic incorporation of scientific evidence in healthcare management within the SUS are defended, in order to strengthen practices that consider sexual and gender diversity.

Finally, professional associations, educational institutions, and various organizations and institutions must take an active stance in defending the LGBTQIAPN+ population’s rights, both through public statements and institutional campaigns, as well as through training and monitoring of best practices. Commitment to diversity must be expressed not only in speeches, but in concrete actions that mobilize healthcare professionals and influence the proposal of public policies.
