# Supplementary material for: Sexual and gender diversity in nursing: radicalizing knowledge and practices
Source: Rev Esc Enferm USP. 2026 Mar 2;60:e20250275. doi: 10.1590/1980-220X-REEUSP-2025-0275en (PMC12952795; doi:10.1590/1980-220X-REEUSP-2025-0275en)
Supplement: Translation

# Translation: Diversidade sexual e de gênero na enfermagem: radicalizar saberes e práticas

**DOI:** 10.1590/1980-220X-REEUSP-2025-0275pt

**Authors:** Lucas Cardoso dos Santos, Aurea Tamami Minagawa Toriyama, Lucia Yasuko Izumi Nichiata

**Affiliations:** 1 Universidade de São Paulo, Escola de Enfermagem, Departamento de Enfermagem em Saúde Coletiva, São Paulo, SP, Brasil.; 2 Universidade de São Paulo, Escola de Enfermagem, Departamento de Enfermagem Materno-Infantil e Psiquiátrica, São Paulo, SP, Brasil.

Este ponto de vista propõe uma reflexão acerca da necessidade de radicalizar saberes e fazeres da enfermagem diante da diversidade sexual e de gênero, evidenciando como processos de invisibilidades, silenciamentos e apagamentos estão presentes no campo da saúde. Tal radicalização não deve ser compreendida como ruptura destituída de diálogo, mas como aprofundamento do compromisso ético-político e científico da enfermagem com a justiça social. Sustenta-se em uma perspectiva crítica capaz de tensionar normatividades excludentes. Além disso, defende a urgência do avanço do conhecimento e da consolidação de práticas em saúde que não apenas incluam, mas que reparem injustiças históricas.

A diversidade sexual e de gênero tem sido compreendida de diferentes formas ao longo da história da humanidade, e tem emergido nos debates contemporâneos como objeto de análise por diferentes pesquisadores e movimentos sociais.

A diversidade de gênero abrange distintas possibilidades identitárias para além do “feminino” e “masculino”, englobando outras formas de representar e performar socialmente as expressões de gênero^(1)^. Dessa forma, identidade de gênero é a autopercepção de gênero da pessoa e como ela o expressa para a sociedade. Uma pessoa que tem uma identidade de gênero que corresponde ao sexo declarado ao nascimento pode ser nomeada como cisgênera. Porém, se a pessoa não possui uma identidade de gênero equivalente ao sexo atribuído ao nascimento, ela poderá ser compreendida como uma pessoa transgênero ou não-binária^(1,2)^, entre outras identidades que se distanciam do sistema sexo-gênero imposto ao nascimento^(3)^.

A diversidade sexual, por sua vez, conforma a pluralidade de experiências e manifestações da sexualidade humana^(1)^. As vivências de cada pessoa em relação à orientação afetivo-sexual são interpretadas como o direcionamento da atração física, romântica e sexual em direção a um gênero específico, ou como sendo independentes de gênero e identidade de gênero, bem como a ausência (ou pouca) de atração^(1,2)^. As orientações afetivo-sexuais podem ocorrer entre pessoas do mesmo gênero (homossexualidade), entre gêneros diferentes com os quais a pessoa se identifica (heterossexualidade), entre ambos os gêneros (bissexualidade) e ainda se caracterizar pela ausência ou baixa atração sexual (assexualidade), entre outras manifestações. Isso revela que a sexualidade humana não é fixa nem uniforme, mas sim atravessada por múltiplas formas de sentir, desejar e se relacionar.

Trata-se, em ambas as definições, de diversidade de gênero e sexual, da pluralidade do social, das relações e da coexistência de diferentes características, perspectivas e experiências dentro de um grupo e sociedade. São justamente contrárias à cisheteronormatividade, termo surgido da necessidade de analisar e criticar a ideia de que a heterossexualidade e a identificação com o gênero atribuído ao nascer são a norma social^(4)^. A compreensão desses conceitos como sinônimos e a assunção do sentido cisheteronormativo nas relações sociais impõem violências em diversos processos sociais, principalmente às pessoas que apresentam orientações afetivo-sexuais e identidades de gênero que divergem das normas sociais tradicionais^(5,6)^.

No Brasil, país em que mais pessoas lésbicas, gays, bissexuais, transgênero, *queer*, intersexo, assexuais, pansexuais, não-binárias e outras identidades de gênero e orientações afetivo-sexuais (LGBTQIAPN+) são assassinadas no mundo, com uma morte a cada 34 horas^(7,8)^, cerca de 19 milhões de pessoas se reconhecem como pertencentes a esse grupo, representando 12% da população geral^(6)^. Contudo, dados oficiais sobre essa população não são identificados em diferentes países, revelando que a invisibilidade estatística da população LGBTQIAPN+ é, por si só, uma expressão da determinação social, pois impede que se reconheçam necessidades de saúde deste grupo.

À luz desse cenário, pessoas LGBTQIAPN+ enfrentam piores condições de trabalho e vida, além de índices de violência mais altos, com efeito negativo no acesso à escolaridade e ao trabalho formal e, por consequência, em suas necessidades de saúde^(6,7)^. De tal maneira, os modos de adoecer e morrer da população LGBTQIAPN+ são profundamente histórica e socialmente determinados. As iniquidades vivenciadas pela população LGBTQIAPN+, como estigma, exclusão, violência e negação de direitos, expressões da determinação social, operam como elementos estruturais que produzem vulnerabilidades em saúde^(1,2,6,7^).

Neste ponto, faz-se o alerta que as pessoas que compõem a sigla LGBTQIAPN+ não são um grupo homogêneo em suas necessidades de saúde, mas conformam um grupo identitário contrário às definições tradicionais de heterossexualidade e cisgeneridade, vivenciando a lógica histórica de exclusão e silenciamento de corpos dissidentes. Enfrentar as injustiças impostas a esse grupo exige o reconhecimento da dívida histórica com essa população, construída por séculos de patologização, marginalização, ausência e violência. Esse posicionamento requer mais do que inclusão: requer reparação ativa, com ações afirmativas que enfrentem a herança de exclusão, promovendo o protagonismo dessas pessoas na construção de políticas públicas e na garantia de direitos.

A Política Nacional de Saúde Integral de Lésbicas, Gays, Bissexuais, Travestis e Transexuais, promulgada em 2011, constitui uma das medidas de reparação no âmbito do Sistema Único de Saúde (SUS) para a garantia de direitos, como o acesso a serviços de saúde, educação inclusiva e proteção contra violência e preconceito. Apresenta plano operativo com estratégias para o enfrentamento das iniquidades em saúde estruturado em quatro eixos: “acesso da população de Lésbicas, Gays, Bissexuais, Travestis e Transexuais (LGBT) à atenção integral à saúde”; “ações de promoção e vigilância em saúde para a população LGBT”; “educação permanente e educação popular em saúde com foco na população LGBT”; “monitoramento e avaliação das ações de saúde da população LGBT”^(9)^.

A implantação dessa política está condicionada à atuação articulada de governos nas diferentes esferas, incluindo secretarias estaduais e municipais, conselhos de saúde, e órgãos do Ministério da Saúde. Além disso, a presença ativa da sociedade civil, em suas diversas formas de organização, constitui elemento indispensável para assegurar direitos, reduzir iniquidades e promover o exercício democrático do controle social. Contudo, a efetividade dessa política ainda é limitada pelo desconhecimento e pelos frágeis mecanismos de monitoramento e avaliação que fragilizam a identificação de lacunas, o ajuste de práticas e a inclusão plena da comunidade LGBTQIAPN+ nos serviços de saúde^(10,11)^. Portanto, há necessidade de fortalecer estratégias que articulem financiamento, capacitação profissional e ações intersetoriais, assegurando que os princípios de universalidade, integralidade e equidade do SUS sejam concretamente aplicados a todas as identidades de gênero e orientações afetivo-sexuais.

Pessoas LGBTQIAPN+ têm historicamente sofrido interdições, constrangimentos e violências nos serviços de saúde por estudantes e profissionais de saúde que resultam na propagação de estigmas e preconceitos, no afastamento desse público dos serviços e no aumento das iniquidades em saúde, revelando lacunas significativas na formação em saúde^(12)^. As temáticas da diversidade sexual e de gênero têm sido marginalizadas e/ou pouco enfatizadas nos currículos brasileiros na área da saúde^(13)^. Em especial a enfermagem, maior força de trabalho em saúde no Brasil e no mundo, por sua posição estratégica no trabalho em saúde, tem uma função social fundamental — e ainda pouco explorada — na construção de práticas inclusivas, éticas e humanizadas voltadas às pessoas LGBTQIAPN+^(5,12,14,15,16,17)^.

Nos Estados Unidos da América, o tempo dedicado ao ensino sobre as necessidades específicas de pessoas LGBTQIAPN+ em cursos universitários de enfermagem é estimado em duas horas ao longo de toda a formação^(15)^. No Brasil, esse cenário se repete, em que a abordagem sobre questões de saúde relacionadas à diversidade sexual e de gênero ainda ocorre de forma marginal, quando não ausente^(5,16)^. Essa omissão curricular contribui para a perpetuação de práticas excludentes e para a naturalização da cisheteronormatividade nos espaços formativos.

Na pós-graduação, onde a maioria das pesquisas brasileiras em enfermagem é produzida, uma revisão de dissertações e teses sobre a saúde da população LGBTQIAPN+, tomando o limite temporal de 2011 a 2022, mostrou que a maioria dos estudos buscou elucidar questões sobre o acesso dessa população aos serviços de saúde. Foram identificadas barreiras como resultado de preconceitos, violências, ausência de assistência qualificada e dificuldades de acesso a informações para prevenção e tratamento de doenças. Revelaram-se ainda a ausência de estudos que tinham na autoria pessoas LGBTQIAPN+ de movimentos sociais e a necessidade de pesquisas que avancem na aplicabilidade das teorias de enfermagem na assistência ao referido público^(17)^.

Os achados evidenciam a urgência de aproximar formação, pesquisa e práticas de cuidado das reais necessidades da população LGBTQIAPN+. Nesse sentido, torna-se imprescindível que o campo da formação em saúde incorpore de forma crítica e transversal as temáticas da diversidade sexual e de gênero, em seus diferentes níveis, com o objetivo explícito de realizar práticas de saúde inclusivas, afirmativas e reparadoras, capazes de tensionar normatividades excludentes. Nas ações de extensão universitária, deve-se proporcionar espaços privilegiados para aproximação entre universidade e comunidade, favorecendo o diálogo, a escuta qualificada e a construção coletiva de práticas de cuidado mais sensíveis à diversidade. Na prática assistencial, é necessário investir em estratégias de sensibilização e capacitação permanente das equipes de saúde. Na pesquisa em enfermagem, deve-se contribuir na produção de conhecimento sobre as necessidades de saúde das pessoas LGBTQIAPN+. É imperativo que as pesquisas apoiem as práticas, na perspectiva que valorizem a inclusão e o protagonismo da população LGBTQIAPN+.

Experiências exitosas na formação em enfermagem têm mostrado avanços importantes quando a diversidade sexual e de gênero é incorporada de modo sistemático. O uso de metodologias ativas e a inclusão transversal de conteúdos ao longo dos currículos, associados à participação de pessoas LGBTQIAPN+ nos processos formativos, contribuem para ampliar as práticas mais representativas e sensíveis às necessidades dessa população e as competências dos estudantes^(18,19)^. No âmbito institucional, políticas explícitas de inclusão e o engajamento de lideranças em processos de capacitação e reorganização de serviços têm favorecido ambientes de trabalho mais saudáveis e a melhoria da qualidade do cuidado^(20,21)^. Tais iniciativas evidenciam que a inserção crítica e consistente dessas temáticas não apenas qualifica a formação, mas também transforma os contextos de cuidado e trabalho, reafirmando o compromisso ético-político da enfermagem.

Dessa forma, defende-se que o trabalho da enfermagem é de natureza intrinsecamente política, por envolver a tomada de decisão, a mobilização de recursos e a influência nas políticas de saúde, tendo por finalidade a garantia do acesso e a qualidade do cuidado para indivíduos e coletividades. A enfermagem formada por agentes políticos de transformação deve se posicionar de forma ativa no combate ao preconceito institucional contra pessoas LGBTQIAPN+, promovendo ambientes seguros, defendendo políticas de saúde que contemplem as especificidades dessa população e transformando as realidades dos corpos dissidentes da norma.

## CONSIDERAÇÕES FINAIS

Neste ponto de vista, defende-se que o cuidado em enfermagem só pode ser verdadeiramente integral e equânime quando se baseia no respeito às singularidades das pessoas e na escuta ativa de suas vozes. Deve guiar a construção de políticas, pesquisas, saberes, ensino e práticas de cuidado como caminho para a efetiva transformação. Combater o preconceito e reconhecer a dívida histórica de exclusão são passos fundamentais rumo à justiça e reparação.

A diversidade sexual e de gênero como uma dimensão essencial do trabalho em enfermagem deve ser reconhecida como um imperativo ético, político e profissional. Superar as lacunas históricas na formação, enfrentar os preconceitos ainda presentes nas práticas de cuidado e propor novos modos de cuidar exige compromisso, reconhecimento e respeito aos deveres profissionais, especialmente no entendimento de que a identidade de gênero e a orientação afetivo-sexual são elementos importantes do processo de saúde-doença.

Ampliar o trabalho da enfermagem para além da dimensão biomédica nos diferentes níveis de atenção em saúde e educação, por sua capilaridade, com presença constante junto às pessoas, famílias e territórios, desempenha papel estratégico, com potencial transformador e singular nesse processo. Consolidar um trabalho que reconheça, respeite e celebre a diversidade sexual e de gênero é, em última instância, reafirmar os valores fundamentais do respeito à dignidade humana, do compromisso com a vida e da luta por uma sociedade mais justa, plural e diversa. Defendem-se, assim, a relevância da participação da enfermagem na formulação de políticas públicas mais inclusivas e a incorporação sistemática de evidências científicas na gestão e na atenção à saúde no SUS, a fim de fortalecer práticas que considerem a diversidade sexual e de gênero.

Por fim, as entidades de classe, de ensino, e diferentes organizações e instituições devem assumir uma postura ativa na defesa dos direitos da população LGBTQIAPN+, tanto por meio de posicionamentos públicos e campanhas institucionais quanto por ações formativas e de fiscalização das boas práticas. O compromisso com a diversidade deve ser expresso não apenas em discursos, mas em ações concretas que mobilizem os profissionais de saúde e influenciem na proposição de políticas públicas.

# Translation: Diversidad sexual y de género en enfermería: radicalizando conocimientos y prácticas

**DOI:** 10.1590/1980-220X-REEUSP-2025-0275es

**Authors:** Lucas Cardoso dos Santos, Aurea Tamami Minagawa Toriyama, Lucia Yasuko Izumi Nichiata

**Affiliations:** 1 Universidade de São Paulo, Escola de Enfermagem, Departamento de Enfermagem em Saúde Coletiva, São Paulo, SP, Brasil.; 2 Universidade de São Paulo, Escola de Enfermagem, Departamento de Enfermagem Materno-Infantil e Psiquiátrica, São Paulo, SP, Brasil.

Este punto de vista propone una reflexión sobre la necesidad de radicalizar los saberes y las prácticas de enfermería ante la diversidad sexual y de género, poniendo de manifiesto cómo los procesos de invisibilización, silenciamiento y borrado están presentes en el campo de la salud. Esta radicalización no debe entenderse como una ruptura desprovista de diálogo, sino como una profundización del compromiso éticopolítico y científico de la enfermería con la justicia social. Se sustenta en una perspectiva crítica capaz de tensionar las normatividades excluyentes. Además, defiende la urgencia de avanzar en el conocimiento y la consolidación de prácticas en salud que no solo incluyan, sino que reparen injusticias históricas.

La diversidad sexual y de género se ha entendido de diferentes maneras a lo largo de la historia de la humanidad y ha surgido en los debates contemporáneos como objeto de análisis por parte de diferentes investigadores y movimientos sociales.

La diversidad de género abarca distintas posibilidades identitarias más allá de lo “femenino” y lo “masculino”, englobando otras formas de representar y expresar socialmente las expresiones de género^(1)^. De este modo, la identidad de género es la autopercepción de género de la persona y cómo la expresa a la sociedad. Una persona que tiene una identidad de género que corresponde al sexo declarado al nacer puede ser denominada cisgénero. Sin embargo, si la persona no tiene una identidad de género equivalente al sexo asignado al nacer, puede ser considerada como una persona transgénero o no binaria^(1,2)^, entre otras identidades que se alejan del sistema sexo-género impuesto al nacer^(3)^.

La diversidad sexual, por su parte, conforma la pluralidad de experiencias y manifestaciones de la sexualidad humana^(1)^. Las experiencias de cada persona en relación con la orientación afectivo-sexual se interpretan como la orientación de la atracción física, romántica y sexual hacia un género específico, o como independientes del género y la identidad de género, así como la ausencia (o escasa) atracción^(1,2)^. Las orientaciones afectivo-sexuales pueden darse entre personas del mismo género (homosexualidad), entre géneros diferentes con los que la persona se identifica (heterosexualidad), entre ambos géneros (bisexualidad) y también caracterizarse por la ausencia o baja atracción sexual (asexualidad), entre otras manifestaciones. Esto revela que la sexualidad humana no es fija ni uniforme, sino que está atravesada por múltiples formas de sentir, desear y relacionarse.

Se trata, en ambas definiciones, de la diversidad sexual y de género, de la pluralidad de lo social, de las relaciones y de la coexistencia de diferentes características, perspectivas y experiencias dentro de un grupo y una sociedad. Son precisamente contrarias a la cisheteronormatividad, término surgido de la necesidad de analizar y criticar la idea de que la heterosexualidad y la identificación con el género asignado al nacer son la norma social^(4)^. La comprensión de estos conceptos como sinónimos y la asunción del sentido cisheteronormativo en las relaciones sociales imponen violencia en diversos procesos sociales, principalmente a las personas que presentan orientaciones afectivo-sexuales e identidades de género que divergen de las normas sociales tradicionales^(5,6)^.

En Brasil, país en el que se asesina a más personas lesbianas, gays, bisexuales, transgénero, queer, intersexuales, asexuales, pansexuales, no binarias y otras identidades de género y orientaciones afectivo-sexuales (LGBTQIAPN+) en el mundo, con una muerte cada 34 horas^(7,8)^, alrededor de 19 millones de personas se reconocen como pertenecientes a este grupo, lo que representa el 12 % de la población general^(6)^. Sin embargo, los datos oficiales sobre esta población no se identifican en los diferentes países, lo que revela que la invisibilidad estadística de la población LGBTQIAPN+ es, en sí misma, una expresión de la determinación social, ya que impide que se reconozcan las necesidades de salud de este grupo.

En este contexto, las personas LGBTQIAPN+ se enfrentan a peores condiciones de trabajo y de vida, así como a índices de violencia más elevados, lo que tiene un efecto negativo en el acceso a la educación y al trabajo formal y, en consecuencia, en sus necesidades de salud^(6,7)^. De este modo, las formas de enfermar y morir de la población LGBTQIAPN+ están profundamente determinadas por factores históricos y sociales. Las desigualdades que sufre la población LGBTQIAPN+, como el estigma, la exclusión, la violencia y la negación de derechos, expresiones de la determinación social, operan como elementos estructurales que producen vulnerabilidades en salud^(1,2,6,7)^.

En este punto, cabe señalar que las personas que componen el acrónimo LGBTQIAPN+ no son un grupo homogéneo en cuanto a sus necesidades de salud, sino que conforman un grupo identitario contrario a las definiciones tradicionales de hetero-sexualidad y cisgeneridad, que experimenta la lógica histórica de exclusión y silenciamiento de los cuerpos disidentes. Hacer frente a las injusticias impuestas a este grupo exige el reconocimiento de la deuda histórica con esta población, construida por siglos de patologización, marginación, ausencia y violencia. Esta postura requiere más que inclusión: requiere una reparación activa, con acciones afirmativas que hagan frente al legado de exclusión, promoviendo el protagonismo de estas personas en la construcción de políticas públicas y en la garantía de derechos.

La Política Nacional de Salud Integral de Lesbianas, Gays, Bisexuales, Travestis y Transexuales, promulgada en 2011, constituye una de las medidas de reparación en el ámbito del Sistema Único de Salud (SUS) para garantizar derechos como el acceso a los servicios de salud, la educación inclusiva y la protección contra la violencia y los prejuicios. Presenta un plan operativo con estrategias para hacer frente a las desigualdades en salud estructurado en cuatro ejes: “acceso de la población lesbiana, gay, bisexual, travesti y transgénero (LGBT) a la atención integral de la salud”; “acciones de promoción y vigilancia de la salud para la población LGBT”; “educación permanente y educación popular en salud con enfoque en la población LGBT”; “monitoreo y evaluación de las acciones de salud de la población LGBT”^(9)^.

La implementación de esta política está condicionada a la actuación articulada de los gobiernos en los diferentes ámbitos, incluyendo secretarías estatales y municipales, consejos de salud y órganos del Ministerio de Salud. Además, la presencia activa de la sociedad civil, en sus diversas formas de organización, constituye un elemento indispensable para garantizar los derechos, reducir las desigualdades y promover el ejercicio democrático del control social. Sin embargo, la eficacia de esta política sigue estando limitada por el desconocimiento y los frágiles mecanismos de seguimiento y evaluación que dificultan la identificación de las deficiencias, el ajuste de las prácticas y la plena inclusión de la comunidad LGBTQIAPN+ en los servicios de salud^(10,11)^. Por lo tanto, es necesario fortalecer estrategias que articulen el financiamiento, la capacitación profesional y las acciones inter-sectoriales, asegurando que los principios de universalidad, inte-gralidad y equidad del SUS se apliquen concretamente a todas las identidades de género y orientaciones afectivo-sexuales.

Las personas LGBTQIAPN+ han sufrido históricamente prohibiciones, restricciones y violencia en los servicios de salud por parte de estudiantes y profesionales de la salud, lo que ha dado lugar a la propagación de estigmas y prejuicios, al alejamiento de este público de los servicios y al aumento de las desigualdades en salud, lo que revela importantes deficiencias en la formación en salud^(12)^. Los temas de la diversidad sexual y de género han sido marginados y/o poco enfatizados en los planes de estudio brasileños en el área de la salud^(13)^.

En particular, la enfermería, la mayor fuerza de trabajo en salud en Brasil y en el mundo, por su posición estratégica en el trabajo en salud, tiene una función social fundamental —y aún poco explorada— en la construcción de prácticas inclusivas, éticas y humanizadas dirigidas a las personas LGBTQIAPN+^(5,12,14,15,16,17)^.

En los Estados Unidos de América, el tiempo dedicado a la enseñanza sobre las necesidades específicas de las personas LGBTQIAPN+ en los cursos universitarios de enfermería se estima en dos horas a lo largo de toda la formación^(15)^. En Brasil se repite este escenario, en el que el enfoque de las cuestiones de salud relacionadas con la diversidad sexual y de género sigue siendo marginal, cuando no inexistente^(5,16)^. Esta omisión curricular contribuye a la perpetuación de prácticas excluyentes y a la naturalización de la cisheteronormatividad en los espacios formativos.

En los estudios de posgrado, donde se produce la mayor parte de la investigación brasileña en enfermería, una revisión de disertaciones y tesis sobre la salud de la población LGBTQIAPN+, tomando como límite temporal el periodo de 2011 a 2022, mostró que la mayoría de los estudios buscaban esclarecer cuestiones sobre el acceso de esta población a los servicios de salud. Se identificaron barreras como resultado de prejuicios, violencia, falta de asistencia calificada y dificultades de acceso a información para la prevención y el tratamiento de enfermedades. También se reveló la ausencia de estudios cuyos autores fueran personas LGBTQIAPN+ de movimientos sociales y la necesidad de investigaciones que avancen en la aplicabilidad de las teorías de enfermería en la asistencia a dicho público^(17)^.

Los hallazgos evidencian la urgencia de acercar la formación, la investigación y las prácticas de cuidado a las necesidades reales de la población LGBTQIAPN+. En este sentido, es imprescindible que el campo de la formación en salud incorpore de manera crítica y transversal los temas de la diversidad sexual y de género, en sus diferentes niveles, con el objetivo explícito de llevar a cabo prácticas de salud inclusivas, afirmativas y reparadoras, capaces de tensionar las normatividades excluyentes. En las acciones de extensión universitaria, se deben proporcionar espacios privilegiados para el acercamiento entre la universidad y la comunidad, favoreciendo el diálogo, la escucha cualificada y la construcción colectiva de prácticas de atención más sensibles a la diversidad. En la práctica asistencial, es necesario invertir en estrategias de sensibilización y capacitación permanente de los equipos de salud. En la investigación en enfermería, se debe contribuir a la producción de conocimiento sobre las necesidades de salud de las personas LGBTQIAPN+. Es imperativo que las investigaciones apoyen las prácticas, desde la perspectiva de valorar la inclusión y el protagonismo de la población LGBTQIAPN+.

Las experiencias exitosas en la formación en enfermería han mostrado avances importantes cuando la diversidad sexual y de género se incorpora de manera sistemática. El uso de metodologías activas y la inclusión transversal de contenidos a lo largo de los planes de estudio, asociados a la participación de personas LGBTQIAPN+ en los procesos formativos, contribuyen a ampliar las prácticas más representativas y sensibles a las necesidades de esta población y las competencias de los estudiantes^(18,19)^. En el ámbito institucional, las políticas explícitas de inclusión y la participación de los líderes en los procesos de capacitación y reorganización de los servicios han favorecido entornos de trabajo más saludables y la mejora de la calidad de la atención^(20,21)^. Estas iniciativas ponen de manifiesto que la inclusión crítica y coherente de estas temáticas no solo mejora la formación, sino que también transforma los contextos de atención y trabajo, reafirmando el compromiso ético-político de la enfermería.

De este modo, se defiende que el trabajo de la enfermería es de naturaleza intrínsecamente política, ya que implica la toma de decisiones, la movilización de recursos y la influencia en las políticas de salud, con el fin de garantizar el acceso y la calidad de la atención a las personas y las comunidades. La enfermería, formada por agentes políticos de transformación, debe posicionarse de manera activa en la lucha contra los prejuicios institucionales contra las personas LGBTQIAPN+, promoviendo entornos seguros, defendiendo políticas de salud que contemplen las especificidades de esta población y transformando las realidades de los cuerpos disidentes de la norma.

## CONSIDERACIONES FINALES

Desde este punto de vista, se defiende que la atención de enfermería solo puede ser verdaderamente integral y equitativa cuando se basa en el respeto a las singularidades de las personas y en la escucha activa de sus voces. Debe guiar la construcción de políticas, investigaciones, conocimientos, enseñanza y prácticas de atención como camino hacia la transformación efectiva. Combatir los prejuicios y reconocer la deuda histórica de exclusión son pasos fundamentales hacia la justicia y la reparación.

La diversidad sexual y de género como dimensión esencial del trabajo de enfermería debe reconocerse como un imperativo ético, político y profesional. Superar las lagunas históricas en la formación, afrontar los prejuicios aún presentes en las prácticas de cuidado y proponer nuevas formas de cuidar exige compromiso, reconocimiento y respeto por los deberes profesionales, especialmente en el entendimiento de que la identidad de género y la orientación afectivo-sexual son elementos importantes del proceso de salud-enfermedad.

Ampliar el trabajo de enfermería más allá de la dimensión biomédica en los diferentes niveles de atención en salud y educación, por su capilaridad, con presencia constante junto a las personas, las familias y los territorios, desempeña un papel estratégico, con un potencial transformador y singular en este proceso. Consolidar un trabajo que reconozca, respete y celebre la diversidad sexual y de género es, en última instancia, reafirmar los valores fundamentales del respeto a la dignidad humana, el compromiso con la vida y la lucha por una sociedad más justa, plural y diversa. De este modo, se defiende la importancia de la participación de la enfermería en la formulación de políticas públicas más inclusivas y la incorporación sistemática de la evidencia científica en la gestión y la atención sanitaria del Sistema Único de Salud (SUS), con el fin de fortalecer las prácticas que tienen en cuenta la diversidad sexual y de género.

Por último, las entidades profesionales, educativas y diferentes organizaciones e instituciones deben adoptar una postura activa en la defensa de los derechos de la población LGBTQIAPN+, tanto a través de posicionamientos públicos y campañas institucionales como de acciones formativas y de supervisión de las buenas prácticas. El compromiso con la diversidad debe expresarse no solo en discursos, sino en acciones concretas que movilicen a los profesionales de la salud e influyan en la propuesta de políticas públicas.
